# Creating spaces for dialogue: a cluster-randomized evaluation of CARE’s Community Score Card on health governance outcomes

**DOI:** 10.1186/s12913-018-3651-3

**Published:** 2018-11-14

**Authors:** Sara Gullo, Anne Sebert Kuhlmann, Christine Galavotti, Thumbiko Msiska, C. Nathan Marti, Philip Hastings

**Affiliations:** 1 CARE USA, 151 Ellis St NE, Atlanta, GA 30303 USA; 2 College for Public Health & Social Justice, Saint Louis University, 3545 Lafayette Avenue, Salus Center 309, St. Louis, MO 63104 USA; 3 CARE Malawi, Pamodzi House, Private Bag A89, Lilongwe, 3 Malawi; 4 Far Harbor, LLC, 816 Congress Ave #1400, Austin, TX 78701 USA

**Keywords:** Social accountability, Maternal health, Reproductive health, Family planning, Patient satisfaction, Malawi

## Abstract

**Background:**

Social accountability interventions such as CARE’s Community Score Card© show promise for improving sexual, reproductive, and maternal health outcomes. A key component of the intervention is creation of spaces where community members, healthcare workers, and district officials can safely interact and collaborate to improve health-related outcomes. Here, we evaluate the intervention’s effect on governance constructs such as power sharing and equity that are central to our theory of change.

**Methods:**

We randomly assigned ten matched pairs of communities to intervention and control arms, administering endline surveys to women in each arm who had given birth in the last 12 months. Forty-six governance items were reduced by factor analysis into eight underlying scales. We evaluated the intervention’s impact on these constructs using local average treatment effect estimates.

**Results:**

Among intervention-area women who reported a community meeting, we further evaluated the influence of the governance constructs on health-related outcomes: home visit from a community health worker, modern family planning, and satisfaction with health services. A significantly greater proportion of intervention-area women compared to control reported the existence of community groups that provide and facilitate negotiated space between community members and healthcare workers (*p* = .003). Several governance constructs were positively associated with the health-related outcomes. Further, active participation in the intervention was also positively associated with several governance constructs.

**Conclusions:**

CARE’s Community Score Card© facilitated the creation and claiming of effective and inclusive negotiated spaces in which community members and healthcare workers could vocalize service delivery issues and prioritize actions for improvement. We argue that reliable measurement of governance concepts such as power sharing, equity and quality of negotiated space, collective efficacy, and mutual responsibility will enhance our ability to evaluate social accountability interventions and understand the processes by which they affect change.

## Background

**Fig. 1:**
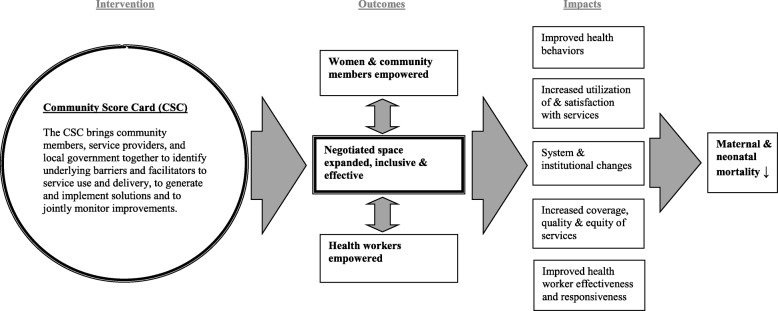
Community Score Card Theory of Change [17]

Social accountability is defined by the World Bank as “…an approach toward building accountability that relies on civic engagement, i.e., in which it is ordinary citizens and/or civil society organizations that participate directly or indirectly in exacting accountability” [1]. The approach seeks to engage citizens in the governance of public sector services and hold governments and health service providers accountable for the quality and equity of these services. Despite general agreement that social accountability involves the intersection of citizen participation and engagement with governance or public sector oversight [1, 2], there have been limited efforts to develop theoretical frameworks for social accountability or to measure its impacts empirically [3, 4]. To address this gap, CARE developed a theory of change and a set of empirical measures for governance concepts that helps unpack this “black box” of what happens in between the implementation of a social accountability approach and health-related outcomes [5]. CARE’s theory of change posits governance outcomes along the casual pathway; these include the empowerment of community members and health workers, plus the creation or claiming of negotiated spaces in which power-holders, health workers, and community members can all interact in a safe, supportive, and equitable environment (see Fig. 1). CARE’s Community Score Card© (CSC) process seeks to facilitate and improve the quality of negotiated spaces so community members and health workers can come together to voice issues, craft solutions, and hold each other mutually accountable for improvement.

Civic participation in governance at the local level can help ensure the equity, quality, and inclusiveness of services, but only if government institutions are willing to engage the public and be responsive to demands [6]. Critical to the success of civic participation and engagement in governance is the composition of who participates, the issues around which they engage [6], and how local government accommodates and responds to this engagement [6, 7]. Although the nature of civic engagement could be adversarial, CARE’s CSC aims to facilitate collaborative engagement between service users and providers. Through this engagement, CARE aims to build relationships and trust and increase power sharing, because interpersonal relationships, trust, and power relations are highly intertwined with patients’ satisfaction and utilization of health services [8–11] and adversarial or oppositional tactics can be detrimental to the long-term goals [12].

Thus, the CSC process intentionally promotes the strengthening of relationships between health workers and community members. The process intends to alter power dynamics and build trust by creating spaces for interaction and negotiation, so that communities and health workers can take mutual responsibility and hold each other accountable for the quality and equity of service provided. Because equity and quality often depend on who participates in negotiated space and how the voices of marginalized groups are heard [13], the CSC process emphasizes the inclusion of women, adolescents, and other frequently marginalized groups. The concerns of both community members and health workers receive equal weight in the CSC process as it strives to improve governance by building trust, power-sharing, collective efficacy, and mutual responsibility between community members and health workers.

In this paper, we use endline data from a cluster-randomized evaluation to test the effect of CARE’s CSC on a set of governance measures that our theory of change suggests will be directly influenced by the intervention and are important milestones along the causal pathway between the CSC intervention and health-related outcomes.

## Methods

### Study setting

Malawi is a heavily rural [14] country of approximately 17.5 million people [15] in southeastern Africa where many families rely on subsistence farming as well as fishing along Lake Malawi. Youth literacy (among 15–24 years) is low among both men (72%) and women (73%) [16]. Despite recent improvements, both maternal and child health indicators are relatively poor. For example, life expectancy at birth is just 55 years, and infant mortality is 46 per 1000 live births [16]. Malawi continues to have one of the highest total fertility rates in world at 5.4, and the maternal mortality ratio is 510 per 100,000 live births [14].

CARE Malawi implemented the CSC intervention, and the corresponding evaluation, in Ntcheu district. The district lies in the Central Region of the country bordering Mozambique. At the time of the intervention, either the Malawi Ministry of Health (MOH) or the Christian Health Association of Malawi (CHAM) served as the administrator of each of the district’s three hospitals and 33 health facilities [17].

### Intervention description

CARE Malawi’s Community Score Card© targeted improvements in maternal and reproductive health-related outcomes, such as family planning, antenatal and postnatal care service utilization, and satisfaction with services. In the intervention areas, CARE Malawi facilitated 26 CSC processes with local health workers and community members in each of the catchment communities surrounding the 10 intervention health facilities. Each cycle of the CSC process included separate meetings with community members and health workers to generate and score indicators, followed by a facilitated interface meeting which brings community members and health workers together with local government officials to discuss their concerns and priorities and to make action plans. Half of the intervention areas completed four cycles of the CSC process by the start of the endline data collection; because the project had a rolling start, the other half completed three cycles (see Gullo and colleagues [17] for a more detailed description of the intervention). CSC participants developed 13 indicators to monitor the CSC process from their perspective and all 13 improved over the course of the intervention [17].

### Evaluation design

We used a cluster-randomized design to evaluate the impact of the CSC intervention on a set of governance-related constructs. Seven of the 33 health facilities in Ntcheu district did not have prevention of mother-to-child transmission of HIV services and were excluded from the sampling frame prior to assignment. The remaining health facilities were matched in pairs based on the presence of basic emergency obstetric services, facility administrator (CHAM or MOH), proximity to the Mozambique border, and population size of the catchment area. Six health facilities did not meet one or more of the criteria for matching, resulting in ten pairs of facilities. One health facility from each pair was randomly assigned to the intervention area; the other was assigned to the control area.

Population data were obtained from government census, district, and local office sources. Intervention facilities contained 290 villages in 56 group villages (GVs) with a total population of 228,029. Control facilities contained 228 villages in 36 GVs with a total population of 170,201. Twenty GVs were selected to participate in the CSC and twenty GVs were selected as control GVs using probability-proportional-to-size (PPS) sampling. Four of the intervention GVs were eliminated due to implementation concerns: participants were thought to be using a different health facility, another maternal and child health project was in the area, or GVs were too close in proximity. Among the 16 included GVs, 64 villages were identified in which CARE Malawi could implement the CSC and 64 villages were randomly selected from the 20 control GVs (see Gullo and colleagues [17] for a more detailed description of the sampling procedures). The primary power analysis conducted prior to the intervention determined the sample size requirements to detect a 10% change in the prevailing rates of institutional births (68%) [18], assuming power = .80, 2-tailed α = .05, non-response = 5%, and design effect = 2.0; the analysis determined that a sample of 650 women per intervention and control would be sufficient to detect this effect.

### Data collection

**Fig. 2:**
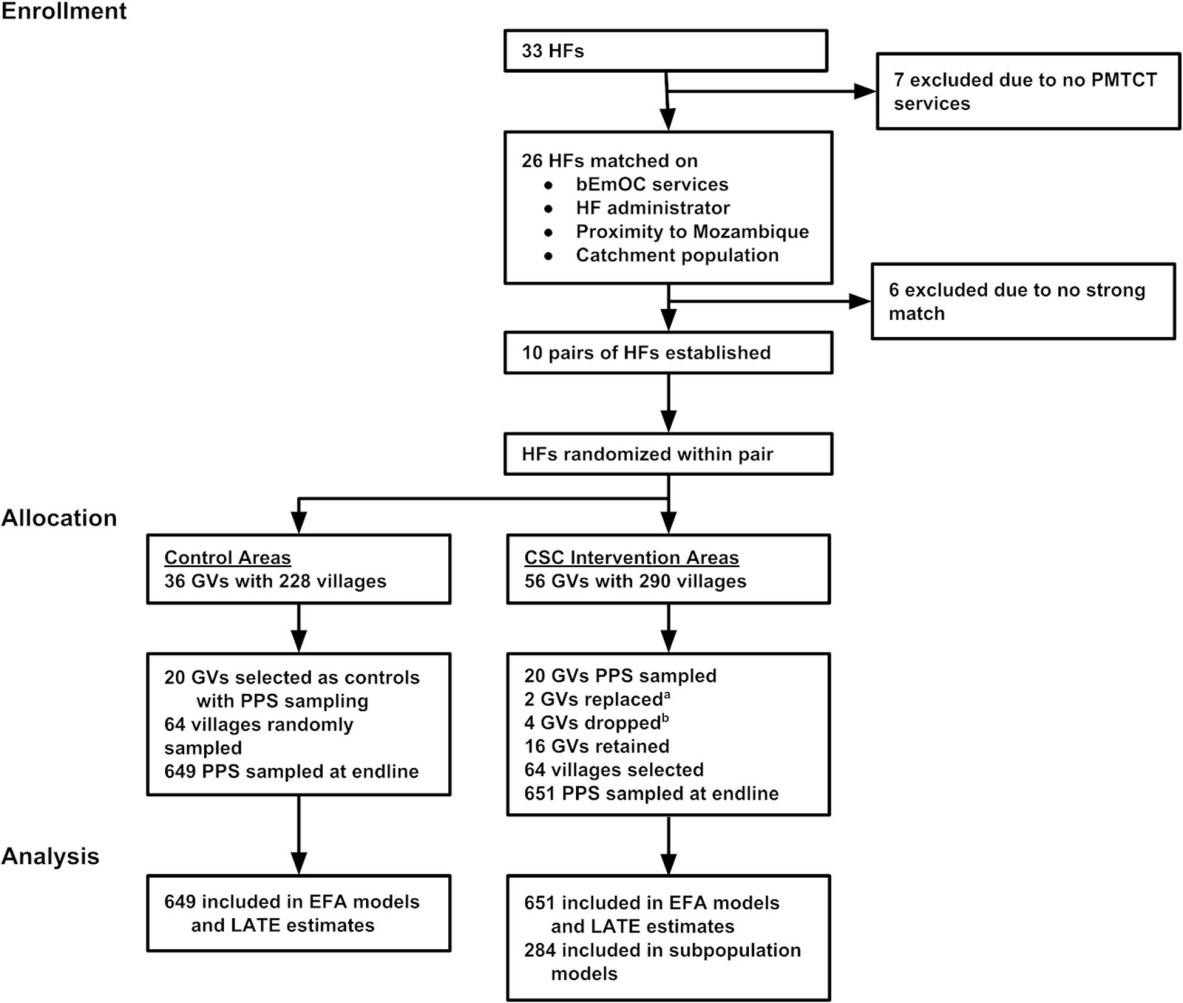
Randomization design flowchart [17]. HF: health facility; GV: group village; PMTCT: Prevention of Mother to Child Transmission of HIV; bEmOC: basic emergency obstetric care. ^a^One GV consisted of a large number of individuals that used a HF in a different catchment area; a second GV was participating in another maternal and child health project. These GVs were replaced with alternative GVs. ^b^Eight GVs were selected from a high population HF, which could not be implemented feasibly within one area. Thus, four GVs were eliminated and the PPS sample for this HF was obtained from the remaining four GVs in the HF catchment area

Cross-sectional surveys were administered at endline to women aged 15–49 who gave birth within the prior 12 months and whose baby was still living. Endline data collection took place in November and December 2014 with 651 women sampled in intervention and 649 in control areas (see Fig. 2). Respondents were selected from every third household. When there was no eligible respondent in the household, interviewers proceeded to the household immediately next door until an eligible respondent was obtained, using a Kish grid in the event of multiple eligible women in a household. All contacted households agreed to participate. The survey took 40–60 min to complete. Malawi’s National Health Science Research Committee reviewed and approved this study as program evaluation. All women provided verbal informed consent prior to the start of the survey.

### Measures

#### Outcomes

Our primary outcomes for these governance-related analyses were a) modern family planning, b) home visit from a community health worker, and c) satisfaction with health services because the CSC had a significant effect on them [17]. Modern family planning was defined as the use of one or more modern family planning methods (i.e., female or male sterilization, oral contraceptive pills, intrauterine device, injections, implant, or male or female condoms) among women who were not pregnant. Home visit from a community health worker assessed visits from a variety of healthcare workers during the respondent’s most recent pregnancy. Satisfaction with services assessed how satisfied respondents were (completely unsatisfied to completely satisfied) with quality of care during antenatal care visit(s), labor and delivery, HIV/AIDS information and services, family planning services, and post-partum visits.

#### Scale and index construction

Forty-six items from the women’s survey pertained to governance. Eight factors were obtained from exploratory factor analysis (EFA) models of groups of similar items. EFA models were fit using Mplus with varimax rotation. The number of factors was determined by eigenvalues greater than one, and items with factor loadings > .4 on a single factor were retained. One item was dropped because it did not meet the factor loading criterion,^1^ two items were dropped due to ambiguous loadings (i.e., comparable loadings on more than one scale),^2^ and one item loaded on unanticipated construct which was an indication of poor validity.^3^ We then constructed scale scores and indexes. Scale scores took the mean; indexes were summed (one point for each yes response). Scale reliability was evaluated using Cronbach’s α with .60–.70 considered acceptable and values greater than .70 considered high reliability.

#### Scales

To assess the relationship between community members and health workers, we measured community members’ *trust in health workers*, the degree to which they perceive health workers as caring, considerate, and attempting to provide the best care possible (see Table 1 for items). Our questions around perceptions of *power-sharing* sought to understand the level of involvement, voice, and influence that community members and health workers each have in decision-making around health care. *Mutual responsibility* measured whether women perceive themselves and health workers together as having an impact on service improvements instead of just one group by itself. As explicit examples of the accountability process, we assessed *joint monitoring and transparency* - measuring whether community members and health workers identify and address concerns - and *equity and quality* – about the breadth of community participation, particularly from women, girls, and other vulnerable groups, and perceptions of the meetings’ inclusivity, content, and management. C*ollective efficacy* assessed women’s confidence in how well community members and health workers could come together to improve services, health status, and the status of women. *Direct outcomes of collective action* and *perceived health outcomes of collective action* assessed perceived improvement in various health care services and procedures resulting from community members and health workers coming together.

**Table 1:** Construct Mean (SE) and Cronbach’s Alpha

Construct	Mean (SE)	Cronbach’s α
Trust in health workers	4.65 (0.02)	.80
I believe the health worker really cares about me		
The health worker is usually considerate of my needs		
I trust the health worker and follow his/her advice		
I trust the health worker’s judgment about my care		
Health workers ensure I get the best possible care		
I trust health workers will keep information private		
Power sharing	4.03 (0.05)	.79
Community & health worker have equal power in service delivery decisions		
Community and health workers have equal voice in deciding how to improve services for women and children		
Community can influence decisions that affect health care		
Mutual responsibility	1.18 (0.01)	.65
Impact on making sure that women are treated with respect by health workers		
Impact on making sure that pregnant women have transportation to the hospital during emergencies		
Impact on increasing the number of days a health worker visits your community		
Impact on making sure the poorest & most vulnerable women & children in the community receive care		
Impact on getting funding to improve health services in this community		
Joint monitoring and transparency	0.76 (0.03)	.93
Problems or other issues with health services were discussed		
Community members voiced their concerns about health services		
Health issues of concern to the most vulnerable & marginalized groups (i.e. youth, women) were discussed		
Plans for improving health services were made		
Health workers voiced their concerns about health services		
The District Health Management Team or local authorities shared concerns & provided information on health issues		
Equity and quality (of negotiated spaces)	0.89 (0.02)	.84
At least half of the community attend these meetings		
At least half of those from the community who attended these meetings were women and girls		
Been well run		
Been inclusive of broad participation from the community		
Been focused on important issues		
Collective efficacy	4.57 (0.02)	.82
Health workers and community members can work together to improve health services for women and children		
People in your community could work together to improve maternal and newborn health services in this community		
People in your community could work together to improve how women are treated at the health facility		
People in your community could work together to obtain government services and entitlements		
People in your community could work together to improve the health and well-being of women		
Outcomes of collective action (direct)	0.87 (0.02)	.93
Improved the quality of maternal and newborn health services		
Increased the availability of maternal and newborn health services provided in this community		
Improved the level of trust between community members and health workers		
Improved health seeking behaviour for reproductive, maternal and newborn health services		
Improved demand for reproductive, maternal and newborn health services		
Improved referral system for maternity care		
Improved the community access to health-related information		
Perceived health outcomes of collective action	0.62 (0.03)	.92
Do you think the Scorecard process has had an impact on the provision and/or quality of ANC services		
Do you think the Scorecard process has had an impact on the provision and /or quality of Maternity services		
Do you think the Scorecard process has had an impact on the provision and /or quality of PMTCT services		
Do you think the Scorecard process has had an impact on the provision and /or quality of post- partum services		
Do you think the Scorecard process has had an impact on the provision and /or quality of family planning services		

#### Indices

The *negotiated spaces participation* index measured participation in various types of community groups. The *community help* index measured whether women had received help from a Safe Motherhood Committee or Community Action Group during or following pregnancy.

#### Measurement properties

Most of the scales and indices had relatively high means or sums; power sharing, mutual responsibility, and perceived health outcomes of collective action had slightly lower means (Table 1). The reliability of all scales exceeded .70 for Cronbach’s α except for mutual responsibility (α = .65).

### Analyses

All models included the following covariates: Catholic vs. other, Ngoni vs. other, married/living together vs. other, reads full sentence vs. other, number of lifetime live births, nearest health facility providing delivery services (less than 30 min, 30–59 min, 1–2 h, and over 2 h on foot), and wealth index. The wealth index was constructed using the Demographic and Health Surveys (DHS) methodology [19] with a reduced set of variables. Women in the intervention versus the control communities did not differ on these socio-demographic characteristics with the exception of lifetime number of live births, which was slightly greater in the treatment area (*p* = .05), suggesting that the randomization process worked to balance these observed characteristics. All models included weights adjusted for village, GV, and health facility cluster. The complex sampling design was accounted for through standard errors computed in SAS 9.3 and STATA 14.

#### Treatment impact analysis

At endline we found that 25.8% of treatment women reported participation in the CSC, while 5.7% of the control areas reported participation (suggesting measurement error or treatment leakage). Given the discrepancy between the intended and the delivered treatment, an intent-to-treat model would severely underestimate the CSC impact. For these situations, the local average treatment effect (LATE) can estimate the effectiveness of the intervention as if there had been exact correspondence between the intended and the delivered treatment [20, 21]. The LATE adjusts the treatment effect by partialing out the impact of non-adherence to treatment assignment due to both non-compliance in the treatment area (i.e., respondents that did not participate in the CSC) and defiance in the control area (i.e., respondents that did participate in the CSC). Thus, the LATE represents a treatment effect that would be observed with perfect compliance. LATE effects were considered statistically significant for *p* values < .05. One requirement for using the LATE method is that the instrument (i.e., the randomly assigned intervention areas) be unrelated to the outcome except through the delivered intervention (i.e., whether a respondent participated in the CSC). Several outcomes contained skip patterns correlated with the instrument that were not included in LATE models. Therefore, impact of the CSC was estimated using LATE models for the remaining constructs: trust in health workers, power sharing, mutual responsibility, collective efficacy, and the existence of a Community Action Group or Safe Motherhood Committee.

#### Health outcome analysis

Among the respondents in the intervention areas who indicated that meetings between health providers and communities had taken place in their village (*n* = 284), we tested the relationship between governance and three health-related outcomes: modern family planning, home visit from a community health worker during pregnancy, and satisfaction with health services.

## Results

Similar to Malawi’s population overall, approximately 50% of the women in our sample were under the age of 25 years (see Table 2). Less than two-thirds were functionally literate. Approximately one-half had three or more living children. Similarly, about 50% lived over one hour from the nearest health facility with basic emergency obstetric care available. Reflective of the Ntcheu district, nearly all the women were of Ngoni ethnicity and were married or co-habitating with their partner.

**Table 2:** Selected socio-demographic and household characteristics of women who gave birth in the last 12 months: Endline, 2014^a^

Characteristic	Controls N = 649	Treatment N = 651
Age (years) (%)		
15–19	21.5%	16.5%
20–24	32.3%	33.9%
25–29	19.0%	21.0%
30–34	20.2%	18.8%
> =35	7.0%	9.9%
Religion (%)		
Catholic	17.9%	23.1%
Presbyterian	11.3%	9.9%
Other Christian	63.2%	59.8%
Other	7.6%	7.3%
Ethnicity (%)		
Ngoni	88.9%	89.2%
Other	11.1%	10.8%
Marital Status (%)		
Never married and never lived together	6.8%	3.9%
Married/currently living together	87.2%	89.2%
Divorced/separated/widowed	6.0%	6.9%
Reading level (%)		
Cannot read simple sentence	27.0%	31.4%
Can read part of the sentence	9.3%	11.9%
Can read the entire sentence	63.7%	56.7%
Parity (%)		
1	31.0%	25.7%
2	20.8%	22.9%
3–4	33.7%	33.9%
5+	14.5%	17.6%
Nearest health facility providing delivery services (%)		
Less than 30 min on foot	21.3%	23.2%
30–59 min on foot	24.7%	24.8%
1–2 h on foot	34.9%	36.6%
More than 2 h on foot	19.1%	15.3%
Household wealth (mean/SE)^b^	0.05 (0.09)	0.13 (0.08)

Footnotes:

^a^weighted percentages & means

^b^computed from principal components analysis implemented using the Demographic and Health Surveys methodology (Rutstein & Johnson, 2004)

The CSC facilitates the development of local committees or groups that enable community members to share experiences, coordinate their voices, and interact with health workers and local government officials around maternal and newborn health issues of concern to them. In communities where such committees existed prior to the intervention but were not fully functioning, the CSC supported and facilitated the reinvigoration of these committees. At endline, significantly more women in the intervention than the control communities reported the existence of these Community Action Groups or Safe Motherhood Committees (*p* = .003) (Table 3).

**Table 3:** Local average treatment effect (LATE) estimates for selected outcomes

Variable	LATE Estimate	Confidence Interval	*t*	*p*
Trust in health workers	0.11	−0.30 - 0.53	0.54	.590
Power sharing	0.48	−0.41 - 1.37	1.10	.281
Mutual responsibility	0.01	−0.12 - 0.15	0.21	.831
Collective efficacy	0.31	−0.12 - 0.74	1.44	.158
Is there a Community Action Group or Safe Motherhood Committee?	0.94	0.34–1.55	3.16	.003

^a^Estimates were obtained from separate models for each outcome. All models contained CSC participation instrumented on treatment assignment (the LATE effect shown above), and the following covariates: religion, ethnicity, current marital status, literacy, number of lifetime live births, wealth index, and nearest health facility providing delivery services

Among women in the intervention areas who were aware of the CSC intervention, active participation in the intervention was positively associated with 7 of our 13 governance constructs (Table 4), including joint monitoring and transparency as well as equity and quality. However, the trust in health workers and mutual responsibility scales were negatively associated with active CSC participation.

**Table 4:** Selected governance-related outcomes as predicted by participation in CARE’s Community Score Card© (subpopulation analysis^b^)

Outcome	Estimate	Confidence Interval	*t*	*P*
Trust in health workers	−0.11	− 0.22 - -0.00	− 2.04	.049
Power sharing	0.03	−0.27 - 0.32	0.20	.844
Mutual responsibility	−0.09	−0.16 - -0.03	−2.97	.005
Negotiated spaces participation	0.68	0.41–0.94	5.18	<.001
Joint monitoring and transparency	0.10	0.01–0.20	2.29	.029
Equity and quality	0.11	0.06–0.16	4.27	<.001
Collective efficacy	−0.07	−0.22 - 0.08	−0.99	.331
Outcomes of collective action (direct)	0.13	0.07–0.20	4.02	<.001
Perceived health outcomes of collective action	0.24	0.12–0.35	4.34	<.001
Is the HAC an effective bridge between the health facility and community?	−0.00	−0.08 - 0.07	−0.09	.925
Is there a Community Action Group or Safe Motherhood Committee?	0.15	0.07–0.24	3.70	<.001
Does Community Action Group or Safe Motherhood Committee provide maternal and newborn health support?	−0.02	−0.06 - 0.03	− 0.70	.490
Community help	0.26	0.04–0.48	2.44	.020

^a^Estimates were obtained from separate models for each outcome. All models contained the following covariates: religion, ethnicity, current marital status, literacy, number of lifetime live births, wealth index, and nearest health facility providing delivery services

^b^Among those women in the intervention areas who reported a meeting in their community

We further analyzed these women to evaluate the relationship between our governance measures and health-related outcomes. Several of the governance measures were significantly associated with one or more of the health-related outcomes. Eight of our 13 governance measures were significantly associated with receiving a home visit from a health worker (Table 5). Four of our governance measures that predicted home visits were also significantly associated with modern family planning use (Table 6). While six of our 13 governance measures were significantly associated with service satisfaction, four of these were negative associations indicating that women who actively participated in the intervention were more *dis*satisfied with the services they received (Table 7).

**Table 5:** Home visit from health workers as predicted by governance-related measures (subpopulation analysis^b^)

Independent variable	Odds Ratio	Confidence Interval	Χ^2^	*p*
Trust in health workers	1.79	1.10–2.92	5.45	.020
Power sharing	1.14	0.82–1.58	0.61	.434
Mutual responsibility	1.20	0.58–2.48	0.24	.623
Negotiated spaces participation	1.64	1.31–2.06	18.77	<.001
Joint monitoring and transparency	1.93	1.04–3.56	4.37	.037
Equity and quality	2.94	0.85–10.17	2.88	.089
Collective efficacy	1.84	1.12–3.01	5.89	.015
Outcomes of collective action (direct)	5.44	1.94–15.20	10.42	.001
Perceived health outcomes of collective action	1.21	0.73–2.00	0.54	.463
Is the HAC an effective bridge between the health facility and community?	3.26	1.13–9.41	4.77	.029
Is there a Community Action Group or Safe Motherhood Committee?	2.81	1.12–7.04	4.83	.028
Does the Community Action Group or Safe Motherhood Committee provide maternal and newborn health support?	1.49	0.42–5.28	0.37	.541
Community help	3.71	2.07–6.67	19.34	<.001

^a^Estimates were obtained from separate models for each outcome. All models contained the following covariates: religion, ethnicity, current marital status, literacy, number of lifetime live births, wealth index, and nearest health facility providing delivery services

^b^Among those women in the intervention areas who reported a meeting in their community

**Table 6:** Modern family planning as predicted by governance-related measures (subpopulation analysis^b^)

Independent variable	Odds Ratio	Confidence Interval	Χ^2^	*p*
Trust in health workers	1.03	0.53–2.00	0.01	.922
Power sharing	0.98	0.70–1.39	0.01	.927
Mutual responsibility	0.45	0.14–1.40	1.93	.165
Negotiated spaces participation	1.36	0.97–1.90	3.12	.077
Joint monitoring and transparency	2.35	1.22–4.54	6.47	.011
Equity and quality	2.23	0.78–6.38	2.23	.135
Collective efficacy	0.90	0.48–1.71	0.10	.758
Outcomes of collective action (direct)	2.60	1.02–6.64	4.02	.045
Perceived health outcomes of collective action	1.02	0.63–1.65	0.00	.945
Is the Health Advisory Committee an effective bridge between the health facility and community?	3.39	1.51–7.63	8.70	.003
Is there a Community Action Group or Safe Motherhood Committee?	1.41	0.73–2.74	1.04	.308
Does the Community Action Group or Safe Motherhood Committee provide maternal and newborn health support?	1.68	0.37–7.69	0.45	.504
Community help	1.59	1.10–2.31	6.00	.014

^a^Estimates were obtained from separate models for each outcome. All models contained the following covariates: religion, ethnicity, current marital status, literacy, number of lifetime live births, wealth index, and nearest health facility providing delivery services

^b^Among those women in the intervention areas who reported a meeting in their community

**Table 7:** Satisfaction with services as predicted by governance-related measures (subpopulation analysis^b^)

Independent variable	Parameter estimate	Confidence Interval	*t*	*p*
Trust in health workers	0.32	0.18–0.47	4.51	<.001
Power sharing	0.01	−0.02 - 0.04	0.64	.528
Mutual responsibility	0.15	−0.02 - 0.32	1.76	.087
Negotiated spaces participation	−0.03	−0.09 - 0.02	−1.26	.216
Joint monitoring and transparency	−0.08	−0.18 - 0.03	−1.52	.138
Equity and quality	−0.15	−0.25 - -0.05	−3.09	.004
Collective efficacy	0.14	0.03–0.25	2.62	.013
Outcomes of collective action (direct)	−0.14	−0.25 - -0.04	−2.77	.009
Perceived health outcomes of collective action	−0.10	−0.19 - -0.01	−2.20	.035
Is the HAC an effective bridge between the health facility and community?	0.24	−0.12 - 0.60	1.35	.185
Is there a Community Action Group or Safe Motherhood Committee?	−0.10	−0.20 - -0.00	−2.06	.047
Does the Community Action Group or Safe Motherhood Committee provide maternal and newborn health support?	0.10	−0.24 - 0.44	0.60	.554
Community help	0.01	−0.03 - 0.06	0.53	.603

^a^Estimates were obtained from separate models for each outcome. All models contained the following covariates: religion, ethnicity, current marital status, literacy, number of lifetime live births, wealth index, and nearest health facility providing delivery services

^b^Among those women in the intervention areas who reported a meeting in their community

Trust in health workers was significantly associated with home visits (*p* = .02) and satisfaction with services (*p* < .001), but not modern family planning. Collective efficacy was similarly associated with home visits (p = .02) and satisfaction (*p* = .01), but not modern family planning. Joint monitoring and transparency of health services was positively associated with home visits (*p* = .04) and modern family planning (p = .01), but not with satisfaction. Similarly, perceiving the health advisory committees as an effective bridge between the community and the health services was positively associated with home visits (*p* = .03) and modern family planning (*p* < .01), but not with satisfaction. Interestingly, perceiving direct outcomes of collective action was positively associated with home visits (*p* = .001) and modern family planning (*p* = .05), but negatively associated with satisfaction with services (*p* = .009).

## Discussion

To our knowledge, this is the first empirical study that attempts to unpack the “black box” of what happens in between the implementation of a social accountability approach and the resulting reproductive health-related outcomes. Our theory of change suggests that a critical domain is the creation or claiming of negotiated spaces in which power-holders, health workers, and community members can all interact in a safe, supportive, and equitable environment. Therefore, we set out to develop and assess governance constructs in this domain (i.e. power sharing, trust, links between the community and health providers, quality of negotiated spaces etc.). At endline, significantly more women in the intervention than the control communities reported the existence of groups that serve as critical links between the community and facility, Community Action Groups or Safe Motherhood Committees. Our findings are consistent with previous research showing that social accountability approaches can create space for dialogue and negotiation between stakeholders [22, 23].

Among women in the intervention areas who were aware of the CSC intervention, active participation in the intervention was positively associated with 7 of our 13 governance constructs. We documented significant relationships between those who actively participated in the intervention and perceptions of equity and quality of the negotiated space. While this measure focuses on perceptions of equity, we need to do more work in the future around understanding who has a voice, who participates in these platforms, and whose voice gets taken into account in social accountability platforms such as the CSC. We also found positive relationships with governance measures of actions resulting from the negotiated space such as joint monitoring and transparency, collective action, and availability of community help.

Interestingly, however, we found CSC participation negatively associated with trust in health workers despite other evidence that the CSC improved relationships between health workers and community members [17]. Program documentation and discussions revealed some unfulfilled promises made by the District Health Management Team (DHMT) (i.e. promising an ambulance that had not yet been delivered) lowered the trust between the community and health workers. Despite the fact that some actions the DHMT was charged to fulfill (like the ambulance) would require an extended timeline and additional funds. Based on our experience in Malawi, we recommend that the CSC process place more emphasis on sharing local governments’ constraints with the community, on higher-level advocacy, and on setting realistic expectations so that failure to achieve all goals does not damage relationships.

In addition to the CSC’s influence on select governance measures, our results highlight the links between these governance measures and important health-related outcomes that the CSC improved [17]. Over half of the governance measures were positively associated with having received a home visit from a health worker including collective efficacy and trust. We tailored the items in our collective efficacy scale to focus specifically on women’s belief that the community could work together to influence issues around maternal newborn health and improve government-provided services. Others have also documented the importance of collective efficacy as part of the process to mobilize communities and improve governance [24, 25]. Similarly, trust has been cited as critical for success in the social accountability literature [26]. Trust is an important element of the patient-provider relationship, predicting crucial outcomes such as utilization of prevention services and treatment adherence [27, 28].

The four governance measures positively associated with modern family planning, including joint monitoring and transparency and collective action, all reflect actions resulting from the CSC process. Our joint monitoring and transparency scale is an important governance measure because of its ability to gauge perceptions about the ability of all participants – community members, health workers, and local authorities – to voice concerns and make plans to improve services. Similarly, community collective action has been identified as an important influence on health worker accountability [29]. Here, our collective action measure focuses specifically around maternal newborn health concerns.

The relationship between our governance measures and women’s satisfaction with services is more complicated than with home visits or modern family planning as several measures were negatively associated with service satisfaction. Satisfaction is an important outcome for social accountability interventions because a woman’s satisfaction with services drives her health behaviors and service utilization [9, 30]. Women who indicated that there were meetings in which community members, health workers and local government officials discussed problems and made improvement plans were *less* likely to be satisfied with health services than their counterparts. Perhaps these meetings highlighted service problems and areas that needed improvement which in turn negatively influenced women’s perceptions of and satisfaction with services. As a result of participation in these meetings, women may no longer be satisfied with the *status quo* of health services because they know that they have a voice and that there is an opportunity to improve. Importantly, however, trust in health workers was positively associated with service satisfaction, similar to others’ findings [27, 31]. Further research needs to unpack this relationship between governance and satisfaction with services. Given the difficulty in measuring satisfaction accurately, our measure of satisfaction may need further refinement.

A few of our governance measures – power-sharing, mutual responsibility, and Community Action Groups or Safe Motherhood Committees providing maternal newborn support – did not show significant relationships with any of our health-related outcomes. One possibility for this finding is that these measures may reflect long-entrenched power and relationship dynamics between the community and health system that may take longer to change before women in the community perceive that they are on equal footing with health workers. Another possibility is that we may need to refine these measures. Even though the reliabilities were within the acceptable range, power sharing and mutual responsibility did have the lowest Cronbach’s α of all our scales. Mutual responsibility (α = .65) performed nearly identically at baseline (α = .64) [5], suggesting that there is indeed room to enhance measurement of this concept. Through the CSC process, the role of the community, health workers, and the DHMT is clarified. Perhaps, the CSC process clarified for women legal responsibility for various aspects of care and where ultimate responsibility lies, but these nuances around women’s perception of power sharing and mutual responsibility need to be refined.

### Limitations

Our results should be interpreted within the context of some real-world evaluation limitations. First, we accelerated our endline data collection by 12 months because other non-governmental organizations were anxious to expand maternal newborn health services into the control communities. This condensed timeframe meant that intervention communities completed fewer cycles of the CSC than originally planned for endline data collection. Therefore, communities received a less intense intervention dose than anticipated, which may have contributed to fewer significant relationships between the CSC and governance measures than hypothesized. Second, several of the governance concepts might require a longer timeframe to observe change as they are, ultimately, changes in relationships. Third, the importance of context specificity for social accountability interventions may limit the generalizability of some of our findings. Next, the matching process ultimately resulted in a larger population in the treatment catchment areas than in the intervention areas. This discrepancy could either increase or decrease the intervention’s effectiveness depending on moderating factors such as how the intervention functions in more densely populated areas. Finally, the “cash-gate” scandal hit Malawi during the height of the intervention period [32], freezing funds from external donors and creating shortages of supplies and personnel within the health system. The intervention may actually have buffered the intervention communities from the worst repercussions of the scandal despite interruptions to supply chains and payments [17].

## Conclusions

The CSC process appears to influence key governance-related constructs that we theorize are important milestones (or causal links) between the intervention and major health outcomes. CARE’s CSC intervention enhanced these governance constructs by creating and facilitating safe, inclusive, and equitable negotiated spaces. Community members, health workers, and local government officials collaborated within these spaces to achieve higher-quality, more transparent, and more accountable health services. Active participation in the CSC ensures a safe, inclusive space to voice concerns and work together to improve health services and outcomes. These spaces enable interpersonal exchanges between community members and health workers so that together they can affect change. In order to understand the true impact of social accountability and how it works to improve health outcomes, we suggest measuring the governance processes affected by such interventions. We also need continued research to measure the sustainability of the intervention and these outcomes, especially after CARE steps back from its facilitation role in the CSC. We encourage those who evaluate social accountability programs to employ similar governance measures; knowledge of how these interventions change the relationships between community members and health workers can, ultimately, help to improve health services.

## Abbreviations

CHAM: Christian Health Association of Malawi
CSC: Community Score Card
DHMT: District Health Management Team
DHS: Demographic and Health Surveys
EFA: Exploratory Factor Analysis
GV: Group Village
LATE: Local Average Treatment Effect
MOH: Ministry of Health
PPS: Probability-Proportional-to-Size

## Footnotes

1 “Health Workers have influence over decisions about the provider of health services”

2 “Meetings in which — Information about health services was shared?” and “Community and health care providers together — Mobilized resources, including in-kind and financial, to improve health services?”

3 “Satisfied with the amount of influence of Health Workers not included in scale scores”

## References

[1] World Bank (2003). World Development Report 2004: Making services work for poor people.

[2] Fox J. Social Accountability: What Does the Evidence Really Say? Global Partnership for Social Accountability. Working Paper No1. 2014. http://gpsaknowledge.org/wp-content/uploads/2014/09/Social-Accountability-What-Does-Evidence-Really-Say-GPSA-Working-Paper-1.pdf Accessed 7 May 2018.

[3] Gaventa J, McGee R (2013). The impact of transparency and accountability initiatives. Development Policy Review.

[4] Joshi A (2013). Do they work? Assessing the impact of transparency and accountability initiatives in service delivery. Development Policy Review..

[5] Sebert Kuhlmann A, Gullo S, Galavotti C, Grant C, Cavatore M, Posnock S (2017). Women’s and health workers’ voices in open, inclusive communities and effective spaces (VOICES): measuring governance outcomes in reproductive and maternal health Programmes. Development Policy Review..

[6] Gaventa J, Hickey S, Mohan G (2004). Towards participatory governance: assessing the transformative possibilities. Participation: from tyranny to transformation.

[7] Ravindra A (2004). An assessment of the impact of Bangalore citizen report cards on the performance of public agencies. Evaluation Capacity Development Working Paper.

[8] Sibamo EL, Berheto TM (2015). Community satisfaction with the urban health extension service in South Ethiopia and associated factors. BMC Health Serv Res.

[9] Jallow IK, Chou YJ, Liu TL, Huang N (2012). Women's perception of antenatal care services in public and private clinics in the Gambia. Int J Qual Health Care.

[10] d'Ambruoso L, Abbey M, Hussein J (2005). Please understand when I cry out in pain: women's accounts of maternity services during labour and delivery in Ghana. BMC Public Health.

[11] Mohan D, Gupta S, LeFevre A, Bazant E, Killewo J, Baqui AH (2015). Determinants of postnatal care use at health facilities in rural Tanzania: multilevel analysis of a household survey. BMC pregnancy and childbirth.

[12] Gaventa J, Barrett G (2012). Mapping the outcomes of citizen engagement. World Dev.

[13] Murthy RK, Klugman B (2004). Service accountability and community participation in the context of health sector reforms in Asia: implications for sexual and reproductive health services. Health Policy Plan.

[14] World Health Organization (WHO) (2016). Malawi: Country Statistical Profile; 2015.

[15] World Health Organization (WHO). Malawi: Country Statistical Profile; 2018*.* Geneva: WHO; 2015 [cited]. Available from: http://www.who.int/countries/mwi/en/

[16] UNICEF (2017). State of the World’s Children: 2017 Country statistical tables.

[17] Gullo S, Galavotti C, Sebert Kuhlmann A, Msiska T, Hastings P, Marti N (2017). Raising the score: evidence from a cluster-randomized trial in Malawi on the effects of a social accountability intervention on reproductive health-related outcomes. PLoS One.

[18] National Statistical Office (NSO), ICF Macro (2011). Malawi Demographic and Health Survey 2010.

[19] Rutstein SO, Johnson K (2004). The DHS wealth index. DHS comparative reports no. 6. Calverton: ORC Macro.

[20] Angrist JD, Pischke J (2009). Mostly harmless econometrics: an empiricist's companion.

[21] Angrist JD (2006). Instrumental variables methods in experimental criminological research: what, why and how. Journal of Experimental Criminology.

[22] Björkman M, Svensson J (2009). Power to the people: evidence from a randomized field experiment of a community-based monitoring project. Q J Econ.

[23] Zeitlin A, Bategeka L, Guloba M, Kasirye I, Mugisha F. Management and motivation in Ugandan primary schools: impact evaluation final report*.* Center for the Studies of African Economies 2011.

[24] Blankenship KM, West BS, Kershaw TS, Biradavolu MR (2008). Power, community mobilization, and condom use practices among female sex workers in Andhra Pradesh, India. AIDS.

[25] Romero L, Wallerstein N, Lucero J, Fredine HG, Keefe J, O'Connell J (2006). Woman to woman: coming together for positive change-using empowerment and popular education to prevent HIV in women. AIDS Education & Prevention.

[26] Tembo F (2013). Rethinking social accountability in Africa: Lessons from the Mwananchi Programme.

[27] Thom DH, Kravitz RL, Bell RA, Krupat E, Azari R (2002). Patient trust in the physician: relationship to patient requests. Fam Pract.

[28] Thom DH, Hall MA, Pawlson LG (2004). Measuring patients’ trust in physicians when assessing quality of care. Health Aff.

[29] Berlan D, Shiffman J (2011). Holding health providers in developing countries accountable to consumers: a synthesis of relevant scholarship. Health Policy Plan.

[30] Mehta S (2011). Service quality as predicator of patient satisfaction a study of the health care sector. J Health Manag.

[31] Platonova EA, Kennedy KN, Shewchuk RM (2008). Understanding patient satisfaction, trust, and loyalty to primary care physicians. Med Care Res Rev.

[32] The Economist (2014). The $32m heist: Malawi’s "cashgate" scandal.

